# A review of critical brain oscillations in depression and the efficacy of transcranial magnetic stimulation treatment

**DOI:** 10.3389/fpsyt.2023.1073984

**Published:** 2023-05-16

**Authors:** Yi-Chun Tsai, Cheng-Ta Li, Chi-Hung Juan

**Affiliations:** ^1^Institute of Cognitive Neuroscience, College of Health Sciences and Technology, National Central University, Taoyuan City, Taiwan; ^2^Department of Psychiatry, Taipei Veterans General Hospital, Taipei, Taiwan; ^3^Institute of Brain Science, National Yang-Ming Chiao-Tung University, Taipei, Taiwan; ^4^Division of Psychiatry, Faculty of Medicine, National Yang-Ming Chiao-Tung University, Taipei, Taiwan; ^5^Cognitive Intelligence and Precision Healthcare Center, National Central University, Taoyuan City, Taiwan; ^6^Department of Psychology, Kaohsiung Medical University, Kaohsiung, Taiwan

**Keywords:** major depressive disorder (MDD), EEG connectivity, repetitive transcranial magnetic stimulation (rTMS), intermittent theta burst stimulation (iTBS), brain oscillations

## Abstract

Repetitive transcranial magnetic stimulation (rTMS) and intermittent theta burst stimulation (iTBS) have been proven effective non-invasive treatments for patients with drug-resistant major depressive disorder (MDD). However, some depressed patients do not respond to these treatments. Therefore, the investigation of reliable and valid brain oscillations as potential indices for facilitating the precision of diagnosis and treatment protocols has become a critical issue. The current review focuses on brain oscillations that, mostly based on EEG power analysis and connectivity, distinguish between MDD and controls, responders and non-responders, and potential depression severity indices, prognostic indicators, and potential biomarkers for rTMS or iTBS treatment. The possible roles of each biomarker and the potential reasons for heterogeneous results are discussed, and the directions of future studies are proposed.

## 1. Introduction

Major depressive disorder (MDD) is one of the most prevalent mental illnesses worldwide. Currently, approximately 280 million people suffer from depression, according to the statistics of the World Health Organization (1). Additionally, depression is predicted to be in the first rank for causing a global economic burden by 2030 (2). In addition to the pharmacological treatments and psychotherapy, the techniques of non-invasive neuromodulations, such as repetitive transcranial magnetic stimulation (rTMS) and intermittent theta burst stimulation (iTBS), have emerged as effective treatments in depression, which U.S. Food and Drug Administration (FDA) approved in 2008 and 2018, respectively. The antidepressant efficacy of rTMS and iTBS was also evidenced by numerous clinical studies [e.g., (3, 4)]. Nevertheless, more than half of patients with depression do not respond to these non-invasive stimulations, and the neural correlates of TMS in depression remain unclear. For this reason, the growing body of studies focused on the issues of investigating the objective biomarkers for assisting and predicting treatment response in order to elevate the response rate.

Among several approaches, electroencephalography (EEG) is relatively affordable and widely available. Some previous studies have reviewed EEG-based neurophysiological biomarkers for depression for diagnosis or the prediction of the treatment effects, including antidepressant medications and neuromodulations (5–10). These reviews involve power-based, alpha asymmetry, machine learning, evoked potential approaches, etc. Among these variant types of EEG biomarkers, band power and alpha asymmetry results were reported the most (11). In addition, among the band power spectral results, the highest proportion previously proposed were alpha and theta oscillations. However, gamma oscillations have recently become a prominent biomarker for depression (12). In addition to the power-based perspective, depression is considered a network dysfunction disorder, supported by numerous neuroimaging studies such as magnetic resonance imaging (MRI) (13–16). Therefore, indices related to functional connectivity could also be potential biomarkers for depression, whereas such studies were not as numerous as power-based studies.

Given the abovementioned backgrounds, this review focuses on elucidating the role of critical power-based brain oscillations, including alpha, theta, gamma oscillations, and EEG connectivity, which mainly describe cross-frequency coupling in depression. Furthermore, whether these biomarkers could be the prognostic markers of variant forms of rTMS treatments in depression is reviewed. Additionally, the possible roles of each biomarker and the potential reasons for inconclusive EEG markers for depression are also discussed, and the possible approaches for future studies are also proposed.

## 2. EEG and depression

Currently, it is unclear whether pharmacological treatments and brain stimulation treatments will result in different patterns of brain oscillations changing in depression. Before we fully understand the EEG representation in each rTMS and medication treatment, expectations of changes in brain oscillations as non-specific treatment responses' biomarkers were currently followed according to similar logic, despite the fact that the underlying neural mechanisms of these two distinct treatment modalities were different. This review will focus more on EEG predictors for variant TMS with regard to the treatment response EEG indicators for depression. In this review, the relevant original articles are summarized in Supplementary Tables 1–3.

### 2.1. Power based EEG oscillations

#### 2.1.1. Alpha oscillations

Henriques and Davidson (17) pioneered the investigation of cortical activities in depression with resting EEG. In contrast to healthy control participants, lower cortical activities over the left frontal regions represented more alpha activities. In comparison, higher cortical activities at the right, characterized by less alpha, were shown in depression. This phenomenon was known as the frontal alpha asymmetry (FAA) hypothesis in depression. Subsequently, abundant studies investigated the asymmetry of alpha activities, and some are investigated in the occipital or the parietal regions, in depression either compared to healthy control or responders in comparison with non-responders (5, 18–22). Henceforth, the lateralized alpha gradually became the dominant biomarker for depression. However, many studies could not replicate the results of lateralized alpha as a reliable biomarker in discriminating depression (23–27) despite one study further investigating the FAA specifically in female remission depression in comparison with non-remission depression (23). Apart from alpha oscillations in the resting state mentioned above, few studies investigated alpha activities as a biomarker for differentiating MDD under cognitive tasks. For example, Stewart et al. (27) investigated the stronger FAA using a facial emotion task rather than the resting state in MDD. Kustubayeva et al. (28) investigated the alpha lateralization in MDD using a decision-making task, however, specifically only at the feedback stage. From these listed unconcluded results, some researchers have suggested that FAA may not be the perfect diagnostic biomarker and instead may be more focused on the prognostic index (29, 30).

Based on the assumption of frontal activation asymmetry hypothesis in MDD, numerous studies applied TMS, either to activate cortical activity using high-frequency rTMS (≥5 Hz) or iTBS over the left frontal region or left dorsolateral prefrontal cortex (DLPFC) or to inhibit cortical activations using low-frequency rTMS (≤ 1 Hz) or cTBS to modulate the imbalance of frontal cortical activation in depression. The results showed an antidepressant efficacy of TMS in depression. However, the change in FAA or increment of alpha oscillations, if it was considered as biomarkers for TMS antidepressant efficacy after TMS treatment in MDD, was not consistent in previous literature. Some reports aligned with FAA or elevation of alpha activities (31–34), whereas some did not (16, 35, 36). The possible reasons for the heterogeneous results are deliberated in the Section 3. Additionally, one viewpoint has stated that FAA is state-invariant and would remain unchanged following the medication treatment, suggesting that it could be the prognostic biomarker for depression (30), although the effect has not been verified in TMS treatment. The details of this perspective are also described in the Section 3.

In addition to the frontal alpha oscillations, the smaller activities of posterior alpha oscillations were proposed as a potential biomarker for identifying MDD (21, 37, 38). However, few studies did not achieve consistent results (23, 39).

Collectively, FAAs varied results may contribute to their ambiguous significance in differentiating between depression and healthy controls as the declaration in one review (40). Nevertheless, FAA might be a valuable diagnostic biomarker if applied to certain situations, such as emotional tasks (28) or female depression (19). Furthermore, FAA may be more effective as predictive indicators for particular antidepressants, such as selective serotonin reuptake inhibitors (SSRIs) (30). On the contrary, overall resting posterior alpha may be more reliable in differentiating depression and controls. Posterior alpha power is relatively stable under the resting state. Most of the studies reviewed above showed the decreased alpha power in posterior regions in depression seems convincible in comparison with FAA. However, reliability and validity in predicting the treatment response are required for further investigations.

#### 2.1.2. Theta oscillations

Theta oscillations were commonly proposed as another biomarker for depression despite variant indices, such as those found in the anterior cingulate cortex (ACC), theta cordance, and fronto-midline theta. Regarding the diagnosis of depression by resting theta oscillations, rostral ACC (rACC) theta was negatively associated with the Hamilton Depression Rating Scale (HAM-D) scores, which indicated that the higher the rACC theta power, the milder the depressive symptoms (41). In addition, Jaworska et al. investigated larger subgenual ACC (sgACC) theta activity in MDD than in healthy controls (19). Additionally, less frontal theta power was investigated in MDD compared with healthy controls (37). In addition to the resting state, some studies have investigated the biomarkers related to theta oscillations using tasks in depression. For example, Gheza et al. (42) investigated the decrement of fronto-midline theta power in the challenging reinforcement learning task, which indicated deficits in approach motivation in MDD. Furthermore, Dharmadhikari et al. (43) investigated the decreased frontal theta asymmetry during Indian classical music listening compared with not listening in MDD, which showed the reverse pattern in comparison with healthy controls. Furthermore, Koller-Schlaud et al. (44) showed that theta power related to happy faces was higher than to sad faces in bipolar depression; however, the pattern did not show in unipolar depression as well as in healthy controls.

From the review mentioned above, theta oscillations might not be ideal for the diagnosis index of depression due to the varied results and lack of robust evidence. Nevertheless, theta oscillations could be considered antidepressant response predictive biomarkers. Larger resting theta activities in ACC at the pretreatment stage were investigated to predict the antidepressant response, including medication (14, 45–47) and 10-Hz rTMS treatment (48), which was supported by numerous studies. Moreover, one study manipulated the increased frontal ACC theta by asking the participants to perform an rACC-engaging cognitive task between two resting EEG conditions before the rTMS treatment. The results show that the frontal theta activity after the cognitive task at the pre-treatment phase could predict the antidepressant response up to 89% within the area under the curve (AUC) of the receiver operating characteristic (ROC) (49); however, this index could not predict the antidepressant efficacy by delivering iTBS (50). Additionally, the lower relative theta power, which indicated the ratio of theta to total power, in frontal regions at the pre-treatment stage was found in responders than in non-responders, which could predict the antidepressant responses (39, 51).

Theta oscillations also could be the indices for antidepressant efficacy although the role of theta oscillations should be further verified. Theta power evidently increased after rTMS treatment at the frontal (31) or central-posterior site (33). Besides, the increment of frontal theta power after electroconvulsive therapy (ECT) was associated with the treatment efficacy (52). However, other studies could still not replicate the results (35, 36). Additionally, frontal theta cordance, which refers to the calculation involving absolute theta power and relative theta power, was investigated as decreased in the responders after drug treatment (53). Theta cordance was also examined in another research as a predictor of responders by reduction at the first week of 1- or 10-Hz rTMS treatment (54, 55). In addition, regarding task-related indices, higher working memory related to fronto-midline theta power was investigated in responders relative to non-responders after 1 week of 10-Hz rTMS treatment (56, 57) and at the pretreatment stage (56). Recently, Tsai et al. (58) revealed a higher power in theta-alpha amplitude modulation (AM) frequency in responders who were stimulated with prolonged iTBS (piTBS) than that of the sham group. Furthermore, the index was positively correlated with piTBS clinical outcome, which may serve as a potential treatment efficacy predictor of piTBS, especially for treatment-resistant depression in the first 2 weeks.

In short, rACC theta activity seems to be a relatively reliable predictor in determining the treatment efficacy although the results should be acquired from the source-level analysis. Among other theta indices that were calculated by relative power, cordance required more evidence.

#### 2.1.3. Gamma oscillations

Although little attention has been paid to investigating gamma oscillations in human MDD before 2015, gamma oscillations have however emerged as a putative biomarker for MDD, as indicated in a review by Fitzgerald and Watson (12). Few studies presented aberrant gamma oscillations in MDD during the resting state. The enhanced complexity of gamma frequency in the frontal and parietal cortex (59) and greater gamma power in the frontal and temporal regions (60) have been investigated in MDD relative to healthy controls. In addition, the increment of amygdala gamma power was associated with the severity of MDD (61). Another finding in the study of gamma current density showed that healthy individuals with high Beck Depression Inventory (BDI) scores had lower resting gamma activity in the ACC, whereas they had higher gamma activity in the posterior cingulate cortex in comparison with people with low BDI scores (62). In addition, one study showed the lower relative gamma power in the left temporal and bilateral occipital areas in drug-naive first-episode MDD compared with healthy controls. The relative gamma power was negatively correlated with the depressive symptom factors, that is, sleep and cognitive disturbance (63).

Some studies demonstrated the differentiation between groups in gamma oscillations using tasks. Yamamoto et al. (64) presented greater gamma activity over the fronto-central regions in response to emotionally positive words in patients who recovered from MDD in comparison with healthy controls using the emotional identification task. Additionally, MDD displayed enhanced gamma activity over the left anterior temporal region under implicit emotional tasks compared with healthy controls. By contrast, MDD showed smaller gamma power than controls and bipolar depression in the posterior temporal regions during emotional tasks which the authors claimed that gamma oscillations could distinguish between MDD and bipolar depression (65). In addition, MDD was associated with worse inhibitory ability, demonstrated by lower gamma activities in the left motor cortex and pre-supplementary motor area (pre-SMA) in comparison with healthy controls under motor inhibition conditions (66).

The evidence for gamma activity in power-based biomarkers after TMS in MDD is inadequate. A resting-state magnetoencephalography (MEG) study demonstrated an increased gamma power at the left DLPFC after rTMS, which was associated with the improvement of symptoms in MDD (67). Similarly, Noda et al. (68) disclosed that increasing gamma rhythm over a specific frontal site (i.e., F3 electrode) was associated with rTMS therapeutic efficacy under an eye-closed resting state in treatment-resistant depression (TRD). In short, the studies of gamma oscillations as biomarkers for rTMS treatment in MDD were relatively less. The listed two studies reported the relationship between higher gamma oscillations and mitigation of depressive symptoms. This result seemed to contradict most studies described above that higher gamma oscillations were related to severe depression. Undoubtedly, the role of gamma oscillations in depression as well as in rTMS treatment response requires further investigations.

Summarizing the currently limited studies, a trend of higher gamma oscillations in MDD than in controls was shown. However, contradictory findings were also reported. More evidence of the role of gamma oscillations in the diagnosis of MDD and the treatment response remains to be explored.

### 2.2. EEG connectivity

Convergent evidence from abundant neuroimaging studies demonstrates that MDD could be a network-based disorder. The aberrant connections between different regions of the brain across certain frequency bands might lead to depression symptoms. In order to investigate the possible biomarkers that were relatively available for clinical applications, an increasing number of studies began to explore EEG connectivity in MDD.

One study showed that theta connectivity in anterior regions and alpha connectivity over anterior and posterior regions were impaired in depression compared with healthy controls during the resting state (69). Additionally, the higher global coherence in delta, theta, alpha, beta (70), and gamma oscillations (71) was found in depression than in healthy controls. Furthermore, a recent study reported frontolimbic and fronto-central hypergamma connectivity in MDD in contrast to controls. In addition, this gamma over-connectivity was associated with the severity of depression (72). Furthermore, lower beta–gamma coupling in MDD was reported in a recent study, and the beta–gamma coupling was negatively correlated with cognitive disturbance (63). Under the task circumstances, one study revealed a decrement in delta connectivity with an increment in theta, alpha, and beta connectivity in MDD by evaluating phase synchronization during a visual odd-ball task (73). Additionally, depressed patients showed impaired inhibitory control while executing a Go/Nogo task, which was represented by lower connectivity in the gamma band between pre-SMA and right inferior frontal gyrus (rIFG) compared with controls (66).

**Figure 1:**
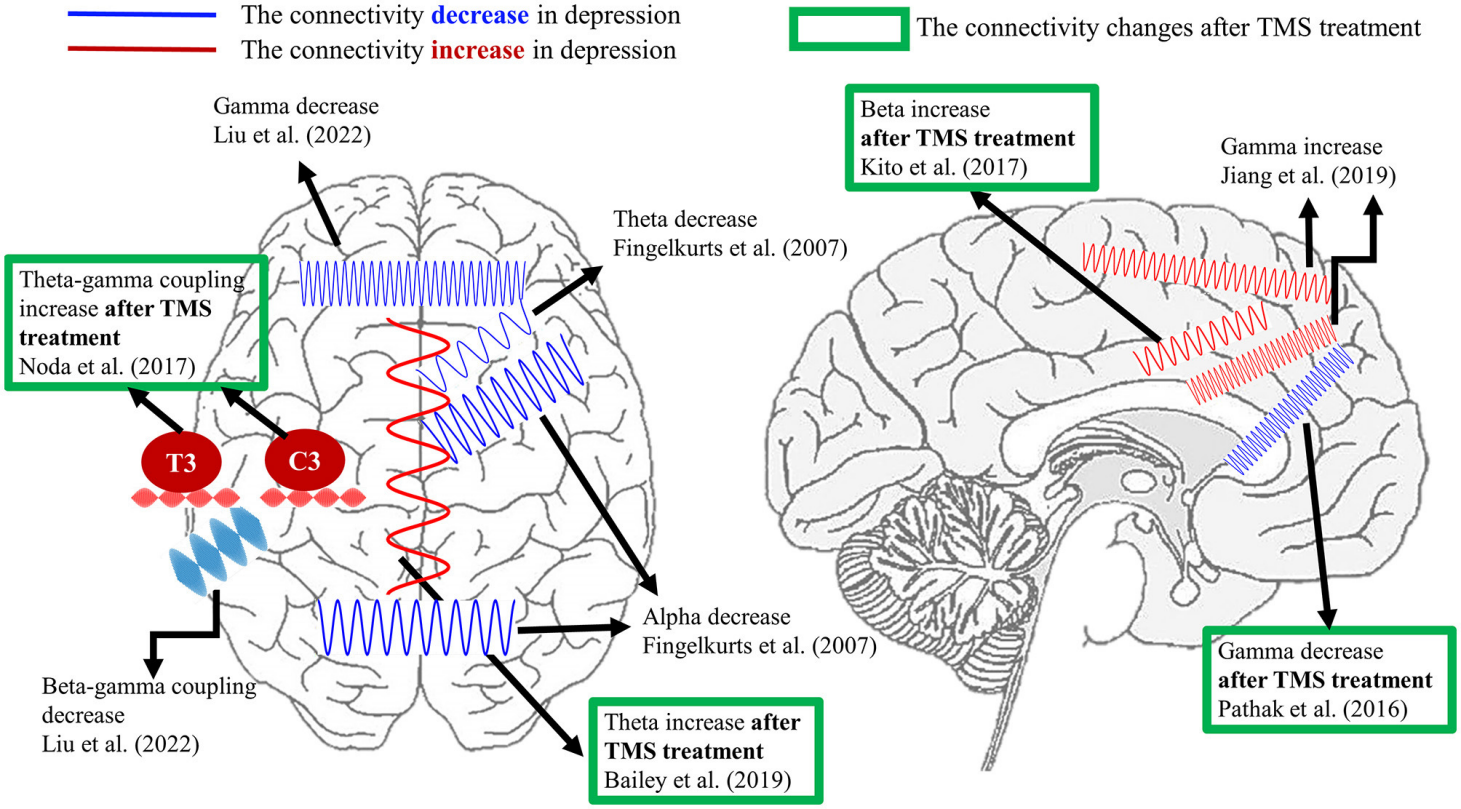
A summary of EEG connectivity in the resting state listed in the present review. The demonstrated oscillations in red color indicate the increment of connectivity between certain brain regions in depression compared with healthy controls. On the contrary, the schematic oscillations in blue color refer to the reduction in connectivity between the illustrated brain areas in depression in comparison with healthy controls. The results marked by the green frame represent the connectivity changes after TMS treatment in depression. Currently, the aberrant theta, alpha, and gamma connectivity were proposed the most in depression. Furthermore, the reported depressive-relevant brain regions were the frontal regions and frontolimbic associations.

Regarding the brain connectivity changes after TMS treatment, Bailey et al. (74) disclosed that the resting theta connectivity could differentiate the responder and non-responder after 1 week of rTMS treatment. Nevertheless, the same group of authors recently reported conflicting results from a larger dataset. The authors claimed that theta connectivity might not be a stable predictor of rTMS treatment in depression (75). Additionally, Kito et al. (76) found an increment in middle beta connectivity between left DLPFC and limbic regions after rTMS treatment in depression by non-linear connectivity analysis. For the gamma connectivity results, resting-state EEG/MEG studies demonstrated the reduction in gamma connectivity between left DLPFC and sgACC (67) as well as precuneus (77) after rTMS in depression. Additionally, the reduced gamma connectivity between left DLPFC and sACC was positively correlated with the improvement of symptoms in MDD (67). Moreover, Noda et al. (68) displayed that the resting theta–gamma coupling at electrodes C3 and T3 was enhanced following a 2-week rTMS treatment. However, the increased resting theta-gamma coupling was not associated with improving depressive symptoms but was correlated with the reduction of error in cognitive performance. For the task-related study, Bailey et al. (56) showed that greater working memory was related to gamma connectivity from baseline to the first week of rTMS in responders than non-responders. The current literature does not show robust and consistent results regarding certain connectivity as a biomarker in the diagnosis or prognosis of depression, which requires more studies. Figure 1 summarizes the current connectivity results during the resting state and the connectivity changes after the TMS treatment listed in this review.

## 3. Discussion

The present review summarized the EEG biomarkers in depression that have been frequently proposed, that is, alpha and theta oscillations, and also the most recently updated gamma oscillations and EEG connectivity. Furthermore, the possible predictors for the antidepressant efficacy of rTMS treatment in depression were summarized. By understanding the present status, the precision of rTMS treatment in depression is expected to be improved in the near future.

Overall, EEG could play a role in predicting the antidepressant response to rTMS, however, due to the complexity of depression, there are solely no best predictors. The validity of the predictors or other possibilities of indices should be confirmed by more evidence. The possible roles of each primary biomarker reviewed in this article, the potential reasons for heterogeneous results, and the approach for future studies are discussed as follows.

### 3.1. The possible roles of each biomarker

Each explored EEG index must have the underlying mechanisms and the possible implications in depression, as well as the effect of rTMS treatment regardless of their reliability and validity. In addition, each index, including each frequency oscillation and connectivity measure, is expected to be state-dependent which indicated that the aberrant EEG characteristics in depression should approximate the pattern in healthy controls following treatment. However, FAA has been considered state-invariant from a recently proposed point of view (30). According to van der Vinne et al. (30), responders displayed right-sided FAA, which refers to larger right frontal alpha than the left, while non-responders showed left-sided FAA both at baseline and after 8 weeks of medication, i.e., SSRI but not Venlafaxine, treatment. The authors demonstrated the trait-like feature which could be a stable predictive prognostic biomarker while not an ideal biomarker for diagnosis (29). Nevertheless, it needs to be further examined whether the characteristics of FAA state-invariant appear in TMS treatment. In addition, whether other indices such as theta oscillations and gamma oscillations that are all state-dependent should be determined as well. Additionally, the FAA index in depression was further explained by Davidson and Henriques (78), that higher right frontal activities might be associated with withdrawal-related emotion while hypoactivation at the left frontal may reflect the insufficiency in approach mechanisms. This implied that the FAA index may be reliable using tasks that elicit emotion, which was not readily shown in the resting state.

Regarding theta oscillations, some studies have demonstrated that the scalp-recorded frontal theta rhythm could reflect the activity in the ACC (79, 80). ACC plays an essential role in emotional regulation, acting as a hub connection between the prefrontal cortex and limbic system (81). In depression, fronto-cingulate dysfunction (14, 45) and maladaptive emotional regulation (82, 83) have been found in various studies. Therefore, the alteration of the theta ACC could reflect fluctuations within the emotional processing ability in depression. Nevertheless, similar to the FAA, the reliability and validity of frontal theta as a biomarker remain inconclusive and require further evidence (84).

Gamma oscillations were rarely discussed in previous studies. However, with the advancement of analytical methods, gamma oscillations have been proposed as another potential biomarker in depression. Gamma oscillations were reportedly associated with inhibitory function, which involved the GABAergic system (85). In depression, abundant evidence advocates that cortical inhibition deficits may give rise to the dysregulation of emotion and cognition (86). From the molecular level to magnetic resonance spectroscopy (MRS) neuroimaging studies, GABAergic interneurons in size as well as density and GABA level were lower in MDD contrasted to the comparison groups (87–90). Several studies showed the increment of GABA levels after rTMS treatment in MDD with MRS measures, which may indicate the improvement of cortical inhibitory function in association with mitigating symptoms (91, 92). However, more studies are required to provide converging evidence regarding changes in GABA levels, gamma oscillations, and improvement of symptoms of depression. These may confirm the role of gamma oscillations as predictors in depression.

Based on the concept of network dysfunction in depression, merely one single frequency band may not fully represent the characteristics of depression (12). The interactions between different frequency bands, such as cross-frequency coupling (CFC), may play a role in transmitting and integrating the information between local cortical processing and long-range brain functions (93). Currently, though less evidence regarding CFC in depression is studied, theta–gamma coupling was mainly reported (94). Theta–gamma coupling was thought to be closely related to information processing and signal integration, which was verified by numerous working memory studies that higher theta–gamma coupling could represent better performance in working memory (95). Depression has been associated with deficits in cognitive functions such as working memory (96) and executive function (97). This might be a source of why theta–gamma coupling could be an indicator of depression. However, the associated underlying mechanisms of theta–gamma coupling in the resting state remain unclear. Furthermore, other possible combinations of connectivity across frequencies, phases, and brain areas, that is, alpha–gamma coupling and delta–gamma coupling, in depression should be explored.

### 3.2. Potential reasons for heterogeneous results

From the review mentioned above, there are no specific biomarkers that can be applied for diagnosis and predicting the rTMS antidepressant efficacy across all patients. Several potential reasons and limitations can cause the heterogeneous results. The first and main overpowering reason is the large heterogeneity of depression *per se*. Second, the different subgroups of depression such as bipolar depression and treatment-resistant depression are also foremost factors. For instance, gamma power could differentiate between bipolar depression and unipolar depression; however, it may not be a suitable index to distinguish between unipolar depression and healthy controls (44). Third, comorbidities would also affect the EEG outcome. Nusslock et al. (98) investigated the alpha asymmetry in depression without anxiety disorder compared with healthy controls; however, this index disappeared in depression with anxiety comorbidities in comparison with healthy controls. Fourth, the interaction of age, gender, and severity of depression are proposed covariant factors in determining valid biomarkers in depression (29). Fifth, the status during the EEG examination, such as whether it is a resting state or a task performance with or without emotional stimuli as reviewed in this article, is another factor to consider. Furthermore, under the resting state, another potential factor that might alter brain oscillations is the issue of vigilance, which denotes the arousal level of the brain. Typically, the vigilance stages and the corresponding EEG frequencies may decrease followed by the passage of time in healthy controls. However, MDD displayed hyperstable vigilance across time and lingered longer in the most alert stage (99, 100). However, the stimulation over the frontal cortex has been demonstrated to decline vigilance (101), and the latency of MDD switching to the next stage probably might be different. Therefore, the investigation of EEG biomarkers corresponding to the TMS effects might depend on the length of the EEG recording; for example, the EEG results of a 2-min recording vs. a 15-min recording may be varied. Additionally, whether or not patients were taking medication, such as lithium, at the time of the EEG recording is another influential factor (102). Additionally, the sample size is also an issue as larger sample sizes may decrease the inter-individual variability.

Apart from these aspects, the limitation of analytical methods also affects the results. Furthermore, other factors, such as the variant EEG montage, anatomical location, preprocessing steps, and the determination of frequency bands, would be important as well (12, 103). Brain EEG signals are composed of linear, non-linear, and non-stationary components, and previous analytical methods based on linear assumptions may limit the comprehensive signal parsing, which was the consensus (104–106). Among several non-linear analytical methods, Huang et al. (107) introduced the Holo-Hilbert spectral analysis (HHSA), which provides full informational representations including the carrier frequency (*f* c) and amplitude modulation frequency (*f* am). HHSA is based on 2-fold signal decomposition by empirical mode decomposition (EMD), consisting of the iterative sifting processes that decompose original signals into a number of intrinsic mode functions (IMFs) from higher to lower frequencies (108). By this method, the frequency bands are determined by the characteristics of the signal *per se* instead of arbitrary selection. In addition, the cross-frequency interaction across the whole brain could be demonstrated at the same time. Recently, several lines of research in vision, working memory, and inhibitory control have demonstrated that these analytical methods can reliably reveal the non-linear/non-stationary features of neuronal responses, such as EEG (109–112) and local field potentials (113, 114). The method has also been applicably pioneered in investigating the EEG biomarkers of rTMS antidepressant efficacy in TRD. The entrainment after-effect of prolonged iTBS in responders revealed enhancement in theta-alpha amplitude modulation frequency by HHSA, which could be a potential biomarker for the efficacy of prolonged iTBS in TRD (58). In addition to these reasons, different protocols of rTMS treatment in depression, such as iTBS, 10-Hz rTMS, and continuous TBS, affect the treatment duration. The fact that sham controls are included or not in a study would also influence the exploration of the predictors in the efficacy of rTMS treatment in depression. These multiple factors may interact and give rise to the abovementioned inconsistent findings.

Other three concerns were crucial to mention in addition to the discussion of potential causes resulting in unconcluded results. First, the meta-analysis article by Widge et al. (115) showed the publication bias that negative or weak studies related to EEG response indices in MDD were not published. This could lead to a false sense of efficacy in clinical trials, where the previously reported minor effects might be amplified. Second, a lack of cross-validation has been practiced in previous studies which may give rise to an overestimation of the efficacy of EEG biomarkers. Test–retest reliability and reproducibility are the third critical factors to consider for examining EEG biomarkers for routine clinical use. Taken together, each biomarker seems to be valid in certain circumstances. Future research should focus on examining more specific and finer categories to determine more precise indices for a given depression condition and a certain treatment. Additionally, the negative results are necessarily reported in the articles. Furthermore, as indicated by Widge et al. (115), the independent sample is recommended to preplan to validate the results. A direct and identical experimental design is anticipated to be conducted for this line of research in order to confirm the reproducibility of certain EEG biomarkers, similar to the approach of recent studies in Alzheimer's disease and insomnia [e.g., (116, 117)].

### 3.3. Approach for future works

In terms of the unclear electrophysiological mechanisms of variant TMS treatments in MDD, non-linear analytical methods are ideal approaches to decipher the hidden information in the EEG signals, which may be applied to power analyses, connectivity, or furthermore source-level analyses. Additionally, the electrophysiological evidence of the iTBS effect in MDD is less explored; however, iTBS has been shown to be more efficient than rTMS in antidepressant efficacy (3). Therefore, discovering the role of iTBS in treating MDD is expected to benefit both clinicians and patients. In addition, MDD was thought to ensue from a glutamatergic dysfunction (118) and gamma-aminobutyric acid (GABA) system (86). TMS-EEG methods have revealed the changes of cortical excitatory and inhibitory circuits in some psychiatric disorders [please see Review (119)], as well as in measuring the modulations of various types of TMS in TRD (120). Li et al. (120) investigated the association between rTMS and glutamatergic neurotransmission and found that iTBS regulates GABA-B receptor-mediated inhibition in MDD. Thus, the TMS types that correspond to TMS-EEG indices in MDD are expected to be investigated for further clinical applications in the future.

For the purpose of improving the efficacy of rTMS treatment in depression and elevating the response rate, the adjustment of protocols in rTMS dependent on each individual will be the trend. One study showed that the individual alpha frequency closer to 10 Hz, which is the exact frequency delivered by rTMS, was associated with the improvement of symptoms in depression (121). This may indicate that the closer the distance between the frequency of exogenous stimulation and intrinsic brain oscillations, the larger is the effect induced, and may reflect an alleviation of depressed symptoms (121). Furthermore, if the stimulation in individualized frequency could be delivered at the relevant phase state of patients, the effect would be even more precise. One study delivered rTMS at the EEG peak of instantaneous alpha frequency in TRD by the method of real-time EEG-triggered TMS. The results showed the decrement of resting alpha power over left DLPFC compared with rTMS delivered at a random alpha phase. However, as the authors mentioned, the selection of the target frequency band and phase as well as the corresponding antidepressant efficacy should be further investigated (122). Moreover, closed-loop neuromodulation therapy has been applied in TRD, although it is a case study with deep brain stimulation treatment. The implanted stimulation would be triggered when the symptom severity is raised by detecting the severity-related biomarker, i.e., gamma power in the amygdala. The results showed significant symptom improvement in the TRD individual (61). Combined with the methods of closed-loop and real-time, the dynamic stimulation with the appropriate frequency and strength could reset the endogenous state, which may be expected to enhance the response rate significantly. One additional approach is that after investigating a number of independent EEG indices, which were proposed to be a predictor of specific TMS treatment responses, the regression model may be created to examine the more effective and comprehensive predictors of TMS antidepressant efficacy. In conclusion, the combination of EEG with individualized (EEG- tailored) TMS protocols and the predictive model may pave the way for precision depression treatment in the near future.

## Author contributions

All authors listed have made a substantial, direct, and intellectual contribution to the work and approved it for publication.
