# Supplementary material for: A review of critical brain oscillations in depression and the efficacy of transcranial magnetic stimulation treatment
Source: Front Psychiatry. 2023 May 16;14:1073984. doi: 10.3389/fpsyt.2023.1073984 (PMC10228658; doi:10.3389/fpsyt.2023.1073984)
Supplement: Supplementary file 1 [file Data_Sheet_1.pdf]

Supplementary Materials

Table 1. *Summary of electrophysiological biomarkers in the diagnosis of depression*

Brain oscillations/ connectivity	Article	Subject and Sample size	Age	Severity of depression	State	Analytical methods	Results
Frontal alpha asymmetry (studies that support the hypothesis)	Gotlib (1998)	31 previously depressed<br>16 currently depressed<br>30 healthy controls	[a]N/A<br>(all female)	Inventory to Diagnose Depression (IDD)≧2	Resting state ([b]EO:1-min /per block; [b]EC:1-min /per block; total with 8 blocks)	Fast Fourier transform<br>(epoch: 2-second)	[c]FAA value (8-13 Hz):<br>MDD < controls<br>(FAA value: negative in MDD; positive in controls)
	Kemp et al. (2010)	15 MDD<br>15 healthy controls	[d]M_{MDD}=39.9 y/o<br>$M_{controls}$=42.4 y/o	[e]HAM-D_{17} ≧18	Resting state (EC: 2-min)	Fast Fourier transform<br>(epoch: 4-second)	FAA value (8-13 Hz):<br>MDD < controls<br>(FAA value: negative in MDD; positive in controls)
	Jaworska et al. (2012)	53 MDD<br>(33 without anxiety; 12 with sub-threshold anxiety; 8 with anxiety)<br>43 healthy controls	M_{MDD}=40.5 y/o<br>$M_{controls}$=36.5 y/o	HAM-D_{17} (M~20.8);<br>[f]MADRS > 22	Resting state (EO: 3-min; EC: 3-min)	sLORETA analysis<br>(epoch: 2-second)	FAA value (10.5-13 Hz):<br>MDD < controls<br>(FAA value: negative in MDD; positive in controls)

Table 1. *(cont.)*

Brain oscillations/ connectivity	Article	Subject and Sample size	Age	Severity of depression	State	Analytical methods	Results
Frontal alpha asymmetry (studies that support the hypothesis)	Arns et al. (2016)	1008 MDD (with 6.2%-10.5% anxiety comorbidity) & 336 healthy controls	M_{MDD}=37.8 y/o $M_{controls}$=37.0 y/o	HAM-D_{17} ≧ 16	Resting state (EO: 2-min; EC: 2-min)	eLORETA analyses (epoch: 4-second)	FAA value (8-13 Hz): -**Female** in EC condition: $MDD_{Non-remission}$ < $MDD_{remission}$
	Čukić et al. (2020)	22 MDD (11 in remission, R; 11 in the episode, E) 20 healthy controls	25-68 y/o M_{MDD}=42.4 y/o $M_{controls}$=30.1 y/o	[g]ICD-10: episode (*M*~36.27); remission (*M*~31.73)	Resting state (EC: 3-min)	Nonlinear analysis of EEG complexity: Higuchi's fractal dimension (HFD); sample entropy (SampEn) (epoch: 5-second)	High alpha band power (10-12 Hz), not low alpha band (8-10 Hz), at right frontal (FP2&F8): MDD_E < controls; MDD_R < controls
	Stewart et al. (2014)	143 with lifetime MDD 163 healthy controls	17-34 y/o	N/A	directed facial action (DFA) task	Fast Fourier transform	FAA value (8-13 Hz): MDD < controls
	Kustubayeva et al. (2020)	60 MDD 60 healthy controls	M_{MDD}=26.6 y/o $M_{controls}$=25.4 y/o	[h]IDS (*M*= 39.93)	Decision-making task	Spectral power density (SPD)	FAA value (8-13 Hz): MDD < controls at feedback stage

Table 1. *(cont.)*

Brain oscillations/ connectivity	Article	Subject and Sample size	Age	Severity of depression	State	Analytical methods	Results
Frontal alpha asymmetry (studies incongruent with the hypothesis)	Debener et al. (2000)	15 MDD<br>22 healthy controls	23-64 y/o<br>M_{MDD}=48.5 y/o<br>$M_{controls}$=45.9 y/o	[i]BDI (*M*= 20.8)	Resting state (EO:2-min /per block; EC:2-min /per block; total with 4 blocks)	Fast Fourier transform (epoch: 2-second)	FAA value (8-13 Hz): MDD < controls (However, FAA value showed **positive** in MDD;<br>positive in controls)
	Mathersul et al. (2008)	52 non-clinical depressed tendency<br>52 healthy controls	18-55 y/o<br>M_{MDD}=32.2 y/o<br>$M_{controls}$=34.9 y/o	Depression Anxiety Stress Scales > 7	Resting state (EC: 2-min)	Fast Fourier transform (epoch: 4-second)	FAA value (8-13 Hz): MDD tendency ~ controls (no difference)
	Stewart et al. (2014)	143 with lifetime MDD<br>163 healthy controls	17-34 y/o	N/A	Resting state (EO:1-min /per block; EC:1-min /per block; total with 8 blocks)	Fast Fourier transform (epoch: 2-second)	FAA value (8-13 Hz): MDD ~ controls However, MDD < controls if analyzing by current source density (but, FAA value showed **positive** in MDD)

Table 1. *(cont.)*

Brain oscillations/ connectivity	Article	Subject and Sample size	Age	Severity of depression	State	Analytical methods	Results
Frontal alpha asymmetry (studies incongruent with the hypothesis)	Arns et al. (2016)	1008 MDD (with 6.2%-10.5% anxiety comorbidity) & 336 healthy controls	M_{MDD}=37.8 y/o $M_{controls}$=37.0 y/o	HAM-D_{17} ≧ 16	Resting state (EO: 2-min; EC: 2-min)	eLORETA analyses (epoch: 4-second)	FAA value (8-13 Hz): -MDD ~ controls (no difference)
Posterior alpha oscillations	Jiang et al. (2016)	22 MDD<br>22 healthy controls	M_{MDD}=33.3 y/o $M_{controls}$=29.9 y/o	HAM-D (*M*= 26.4)	Resting state (EC:4-min)	Source analysis: Dynamic Imaging of Coherent Sources (DICS) beamforming method (epoch: 2-second)	-Posterior alpha (8-14 Hz):<br>MDD < controls<br>-Posterior alpha was negatively correlated to the severity of depression
	Čukić et al. (2020)	22 MDD (11 in remission, R; 11 in the episode, E)<br>20 healthy controls	25-68 y/o<br>M_{MDD}=42.4 y/o<br>$M_{controls}$=30.1 y/o	ICD-10: episode (*M*~36.27); remission (*M*~31.73)	Resting state (EC: 3-min)	Nonlinear analysis of EEG complexity (epoch: 5-second)	Posterior alpha power (8-10 Hz):<br>MDD < controls

Table 1. *(cont.)*

Brain oscillations/ connectivity	Article	Subject and Sample size	Age	Severity of depression	State	Analytical methods	Results
Posterior alpha oscillations	Umemoto et al. (2021)	31 female MDD (13 participants reported a secondary anxiety diagnosis) 35 female healthy controls	13-18 y/o M_{MDD}=16.2 y/o $M_{controls}$=15.2 y/o	BDI-II	Resting state (EC: 4-min; EO: 4-min)	Fast Fourier transform (epoch: 1-min)	-Posterior alpha power (8-13 Hz): MDD < controls -Posterior alpha asymmetry value (left-right) MDD < controls - Posterior alpha asymmetry value was negatively correlated with severity of depression
(contradictory results)	Knott et al. (2000)	70 male MDD	M=37.8 y/o	HAM-D_{17} ≧18	Resting state (EC: 20-min)	Fast Fourier transform (epoch: 2.5-second)	The study did not show any alpha (7.5-12.5 Hz) related results
	Arns et al. (2016)	1008 MDD (with 6.2%-10.5% anxiety comorbidity) 336 healthy controls	M_{MDD}=37.8 y/o $M_{controls}$=37.0 y/o	HAM-D_{17} ≧16	Resting state (EO: 2-min; EC: 2-min)	eLORETA analyses (epoch: 4-second)	Groups did not differ in occipital alpha

Table 1. *(cont.)*

Brain oscillations/ connectivity	Article	Subject and Sample size	Age	Severity of depression	State	Analytical methods	Results
ACC theta	Saletu et al. (2010)	60 female MDD<br>30 controls	M=51.1 y/o<br>M_{MDD}=51.1 y/o<br>$M_{controls}$=22~42 y/o	HAM-D	Resting state (EC: 4-min)	LORETA analyses (epoch: 5-second)	[j]rACC theta (6-8 Hz) power was negatively correlated with HAM-D score
	Jaworska et al. (2012)	53 MDD<br>(33 without anxiety; 12 with sub-threshold anxiety; 8 with anxiety)<br>43 healthy controls	M_{MDD}=40.5 y/o<br>$M_{controls}$=36.5 y/o	$HAM\text{-}D_{17}$ (M~20.8);<br>MADRS > 22	Resting state (EO: 3-min; EC: 3-min)	sLORETA analysis (epoch: 2-second)	[k]sgACC theta (6-8 Hz) power:<br>MDD > controls
Frontal Theta	Jiang et al. (2016)	22 MDD<br>22 healthy controls	M_{MDD}=33.3 y/o<br>$M_{controls}$=29.9 y/o	HAM-D (M= 26.4)	Resting state (EC:4-min)	Source analysis: Dynamic Imaging of Coherent Sources (DICS) beamforming method<br>(epoch: 2-second)	Frontal theta (4-8 Hz):<br>MDD < controls

Table 1. *(cont.)*

Brain oscillations/ connectivity	Article	Subject and Sample size	Age	Severity of depression	State	Analytical methods	Results
Frontal Theta	Dharmadhikari et al. (2018)	23 MDD<br>23 controls	M_{MDD}=34.8 y/o<br>$M_{controls}$=29.5 y/o	HAM-D (8-13)	Listening to the Indian music	Fast Fourier transform	MDD showed decreased frontal theta asymmetry (5-7 Hz) power during music listening compared to not listening
	Koller-Schlaud et al. (2020)	33 MDD<br>22 bipolar disorders (BD)<br>32 controls	M_{MDD}=36.5 y/o<br>M_{BD}=37.6 y/o<br>$M_{controls}$=34.3 y/o	HAM-D<br>MDD (*M*=18.6 y/o)<br>BD (*M*=18.5 y/o)	Emotional task	Fast Fourier transform	BD showed higher theta power in happy faces conditions than in sad conditions. The pattern did not show in MDD and controls
Fronto-midline theta	Gheza et al. (2018)	42 MDD<br>60 controls	*M*=41.4 y/o<br>M_{MDD}=41.4 y/o<br>$M_{controls}$=37.9 y/o	HAM-D (*M*= 21.5)	Reinforcement learning task	Fast Fourier Transform & complex Morlet wavelets	Fronto-midline theta: MDD < controls

Table 1. *(cont.)*

Brain oscillations/ connectivity	Article	Subject and Sample size	Age	Severity of depression	State	Analytical methods	Results
Gamma oscillations	Pizzagalli et al. (2006)	34 female healthy controls:<br>17 High BDI<br>17 Low BDI	M_{high}=31.1 y/o<br>M_{low}=29.4 y/o	High: BDI≧18<br>Low: BDI≦6	Resting state (EO:1-min /per block; EC:1-min /per block; total with 8 blocks)	Discrete Fourier transform & LORETA (epoch: 2048-ms)	Gamma current density (36.5-44 Hz):<br>ACC: High BDI < Low BDI<br>Posterior cingulate cortex: High BDI > Low BDI
	Strelets et al. (2007)	20 MDD<br>28 controls	19-57 y/o<br>M_{MDD}=36.0 y/o<br>$M_{controls}$=20.5 y/o	ICD-10	Resting state (EC: 100-second)	Spectral and coherence analysis (epoch: 5-second)	Gamma power (30-40 Hz):<br>MDD > controls
	Akar et al. (2015)	16 MDD<br>15 controls	M_{MDD}=31.1 y/o<br>$M_{controls}$=29.4 y/o	HAM-D (*M*= 22.6)	Resting state (EC: 3-min)	Discrete wavelet transform & fractality analysis	Complexity of gamma (30-60 Hz):<br>MDD > controls
Gamma oscillations	Scangos et al. (2021)	1 MDD case	36 y/o	MADRS=36	Resting state ([l]SEEG) (15-min)	NeuroPace RNS System 、 Dynamic time warping (DTW) (epoch: 30-second)	Amygdala gamma power was positively correlated to the severity of depression

Table 1. *(cont.)*

Brain oscillations/ connectivity	Article	Subject and sample size	Age	Severity of depression	State	Analytical methods	Results
Gamma oscillations	Liu et al. (2022)	43 MDD<br>57 controls	18-49 y/o<br>M_{MDD}=29.7 y/o<br>$M_{controls}$=27.1 y/o	HAM-D_{17} ≧17	Resting state (EO:1-min /per block; EC:1-min /per block; total with 8 blocks)	Fourier Transform (epoch: 4-second)	-Gamma relative power: MDD < controls<br>-Gamma relative power was negative correlated with depressive symptom factors (sleep disturbance and cognitive disturbance)
	Liu et al. (2012)	20 MDD<br>20 BD<br>20 controls	M_{MDD}=33.5 y/o<br>M_{BD}=34.8 y/o<br>$M_{controls}$=34.1 y/o	HAM-D_{17} MDD (*M*=7.9)<br>BD (*M*=6.7)	Emotional task	Morlet wavelet	Gamma (35-55 Hz) during emotional task:<br>MDD < controls<br>BD < controls<br>MDD < BD
	Yamamoto et al. (2018)	18 recovered MDD<br>33 controls	M_{MDD}=21.7 y/o<br>$M_{controls}$=20.0 y/o	BDI-II (*M*= 9.7)	Emotional task	Wavelet transform	Gamma activity (30-70 Hz) during emotional task: recovered MDD > controls

Table 1. *(cont.)*

Brain oscillations/ connectivity	Article	Subject and Sample size	Age	Severity of depression	State	Analytical methods	Results
Gamma oscillations	Han et al. (2020)	31 MDD<br>19 controls	18-45 y/o<br>M_{MDD}=31.0 y/o<br>$M_{controls}$=31.5 y/o	HAM-D_{17} ≧24	Go/No-Go task	Fast Fourier Transform	Gamma activity (30-50 Hz) during inhibitory control task: MDD < controls
Theta connectivity	Fingelkurts et al. (2007)	12 MDD<br>10 controls	M_{MDD}=43 y/o<br>$M_{controls}$=42 y/o	HAM-D_{17} (*M*= 24)	Resting state (EC: 20-min)	Structural synchrony (epoch: 1-min)	Theta connectivity (5-7 Hz):<br>MDD < controls in anterior regions
	Li et al. (2016)	14 MDD<br>19 controls	*M*=31.2 y/o	HAM-D_{17} (*M*= 23.9)	Visual odd-ball task	Phase synchronization	MDD > controls (target dependent)
Gamma connectivity	Jiang et al. (2019)	22 MDD (9 with anxiety)<br>22 controls	M_{MDD}=33.3 y/o<br>$M_{controls}$=29.9 y/o	HAM-D_{17} (*M*= 26.4)	Resting state (EC: 4-min)	dynamic imaging of coherent sources (DICS) approach (epoch: 2-second)	Gamma band (30-48 Hz) connectivity:<br>-MDD > controls<br>-gamma connectivity was associate with the severity of depression

Table 1. *(cont.)*

Brain oscillations/ connectivity	Article	Subject and Sample size	Age	Severity of depression	State	Analytical methods	Results
Gamma connectivity	Han et al. (2020)	31 MDD<br>19 controls	18-45 y/o<br>M_{MDD}=31.0 y/o<br>$M_{controls}$=31.5 y/o	HAM-D_{17} ≧24	Go/No-Go task	Using new approach method proposed by [m]Hipp et al. (2012)	Reduced gamma connectivity between preSMA and rIFG in MDD during the task
Beta-gamma coupling	Liu et al. (2022)	43 MDD<br>57 controls	18-49 y/o<br>M_{MDD}=29.7 y/o<br>$M_{controls}$=27.1 y/o	HAM-D_{17} ≧17	Resting state (EO:1-min /per block; EC:1-min /per block; total with 8 blocks)	[n]PAC	Beta-gamma coupling in the left temporal:<br>-MDD < controls<br>-Beta-gamma PAC was negative correlated with cognitive disturbance

Note

The order of the articles in this table was summarized by the following rules: First is the study result which showed a similar pattern. Second is the state, e.g., resting state. Third is the year of the publications.

[a] N/A indicates not applicable, which might be due to the insufficient information from the articles.

[b] EO indicates eye-opened in the resting state; EC refers to eye-closed in the resting state.

[c] Frontal alpha asymmetry (FAA) value: the calculation of FAA here adopted right frontal alpha power – left frontal alpha power. Therefore, FFA positive indicates greater right alpha power while FFA negative indicates greater left alpha power.

[d] *M* refers to the average of age.

[e] Hamilton Depression Rating Scale (HDRS or HAM-D; Hamilton, 1960). The score > 18 is severe depression (17-item version).

[f] Montgomerye Åsberg Depression Rating Scale (MADRS; Montgomery and Åsberg, 1979). The score in the range of 20-34 is moderate depression. The score > 34 is severe depression.

[g] International Classification of Diseases (ICD).

[h] Inventory of Depressive Symptomatology I (IDS; Rush et al., 1996).

[i] Beck Depression Inventory (BDI) (BDI-II; 1996). The score > 28 is severe depression.

[j] rostral anterior cingulate cortex (rACC)

[k] subgenual anterior cingulate cortex (sgACC)

[l] Stereoelectroencephalography (SEEG)

[m] Hipp JF, Hawellek DJ, Corbetta M, Siegel M, Engel AK. Large-scale cortical correlation structure of spontaneous oscillatory activity. Nat Neurosci (2012) 15(6):884-890.

[n] Phase amplitude coupling (PAC)

Table 2. *Summary of electrophysiological biomarkers in prognosis and prediction of treatment response at pretreatment stage in depression*

Brain oscillations/ connectivity	Article	Subject and Sample size	Severity of depression	Intervention	Treatment duration	Analytical methods	Results
Alpha oscillations	Arns et al. (2016)	1008 MDD (with 6.2%-10.5% anxiety comorbidity)<br>336 healthy controls<br>M_{MDD}=37.8 y/o<br>$M_{controls}$=37.0 y/o	HAM-D_{17} ≧ 16	Three medication groups: Escitalopram; Sertraline; Venlafaxine-XR	8 weeks	eLORETA analyses (resting state: EO: 2-min; EC: 2-min; epoch: 4-second)	-FAA value in **female** before treatment was negative correlated with HAM-D score at week 8<br>-FAA value in f**emale** before treatment was positive correlated with improvement of symptoms
	Van der Vinne et al. (2019)	1008 MDD (with 6.2%-10.5% anxiety comorbidity)<br>*M*=38.3 y/o	HAM-D_{17} ≧ 16	Three medication groups: Escitalopram; Sertraline; Venlafaxine-XR	8 weeks	eLORETA analyses (same as Arns et al., 2016; epoch: 4-second)	FAA value (8-13 Hz) in female<br>**Pre:**<br>Non-responder < Responder (FAA value: negative in Non-responder;<br>positive in responder)<br>**Post: (same as pre)**<br>Non-responder < Responder (FAA value: negative in Non-responder;<br>positive in responder)

Table 2. *(cont.)*

Brain oscillations/ connectivity	Article	Subject and Sample size	Severity of depression	Intervention	Treatment duration	Analytical methods	Results
Alpha oscillations	Bruder et al. (2008)	18 Depression & 18 healthy adults 20-56 y/o M_{MDD}=35.9 y/o $M_{controls}$=31.7 y/o	HAM-D_{21} (M~19.4)	Medication: Fluoxetine	12 weeks	Fast Fourier transform (resting state (EO:2-min /per block; EC:2-min /per block; total with 4 blocks)epoch: 1.28-second)	-Posterior alpha power (7.8 –12.5 Hz) **Pre:** responders > controls -Posterior alpha asymmetry **Pre:** responders (greater alpha over right) > non-responders
rACC theta	Mulert et al. (2007)	20 MDD: 10 Responders (Age:M=48.8) 10 Non-responders (Age:M =45.4)	HAM-D_{17} at pretreatment: Responder (M=29.1) Non-responder (28.8)	Medication: Citalopram or Reboxetine	4 weeks	LORETA analyses (resting state: EC: 5-min; epoch: ~2-second)	rACC theta (6.5-8 Hz) activity is larger in responder than non-responder at pretreatment stage
	Hunter et al. (2013)	22 MDD: 7 Responders (Age:M=32.3) 15 Non-responders (Age:M=43.3)	HAM-D_{17} at pretreatment: Responder (M=19.4) Non-responder M=20.2)	Medication: Sertraline	5 weeks	LORETA analyses (resting state: EC: 20-min; epoch: 2-second)	Higher rACC theta (4-7 Hz) at pretreatment stage could predict the antidepressant efficacy

Table 2. *(cont.)*

Brain oscillations/ connectivity	Article	Subject and Sample size	Severity of depression	Intervention	Treatment duration	Analytical methods	Results
rACC	Pizzagalli et al. (2018)	248 MDD 18-65 y/o	HAM-D_{17} (M =18.4)	Medication: Sertraline or placebo	8 weeks	LORETA analyses (resting state: EC: 4-min; EO: 4-min epoch: 2-second)	Increased rACC theta (4.5-7 Hz) at baseline is related to the improvement of depressive symptoms
sgACC	Narushima et al. (2010)	43 [a]TRD (Age: *M*=62.5)	HAM-D_{17} > 14	10 Hz [b]rTMS at left [c]DLPFC (N=32) & sham (N=11)	Total 10 daily rTMS sessions Total 12000 or 18000	LORETA (resting state: EC: 20-min; epoch: 3-min)	sgACC theta increased in the responders at pretreatment stage
Relative frontal theta	Knott et al. (2000)	70 male MDD (Age: *M*=37.8 y/o)	HAM-D_{17} ≧ 18	Medication: Paroxetine	6 weeks	Fast Fourier Transform (resting state: EC: 20-min; epoch: 2.5-second)	Lower relative frontal theta (3.5-7.5 Hz) power at pretreatment was related to better improvement of the symptoms
	Iosifescu et al. (2009)	82 MDD (Age: *M*=35.9 y/o)	HAM-D_{17} ≧ 16	Medication: SSRIs or venlafaxine	8 weeks	Fast Fourier transform (resting state: EC: 20-30 mins; epoch: 2-second)	Lower relative frontal theta (3.5-7.5 Hz) power was shown in responders at baseline

Table 2. *(cont.)*

Brain oscillations/ connectivity	Article	Subject and Sample size	Severity of depression	Intervention	Treatment duration	Analytical methods	Results
Frontal theta	Li et al. (2016)	36 TRD (Age: *M*=41.7)	$HAM\text{-}D_{17} \geqq 18$	10 Hz rTMS at left [c]DLPFC	Total 10 daily rTMS sessions	Fast Fourier transform (resting state: EC: 3-min)	Cognition-modulated frontal theta (4-8 Hz) at the pretreatment stage could predict the rTMS efficacy

Note

The order of the articles in this table was summarized by the year of the publications.

[a] Treatment-resistant depression (TRD), which is specifically resistant to drug.

[b] Repetitive transcranial magnetic stimulation (rTMS)

[c] Dorsolateral prefrontal cortex (DLPFC)

Table 3. *Summary of electrophysiological biomarkers after treatment in depression*

Brain oscillations/ connectivity	Article	Subject and Sample size	Severity of depression	Intervention	Treatment duration	Analytical methods	Results
Alpha oscillations (studies that alpha might change after the treatment)	Valiulis et al. (2012)	45 TRD (Age: *M*=52.2)	HAM-D_{17}, BDI and MADRS as clinical assessment	10 Hz rTMS at left DLPFC (N=23)<br><br>1 Hz rTMS at right DLPFC (N=22)	Total 10-15 daily rTMS sessions	Fast Fourier transform (resting EC: 10-min; epoch: 2-second	Alpha power (8-12 Hz) increased in central and parietal regions after 10 Hz rTMS treatment<br>-Frontal alpha power (8-12 Hz) increased towards the right hemisphere after 1Hz rTMS treatment<br>-FFA changes toward right was positive correlated with clinical improvement
	Noda et al. (2013)	25 TRD (Age: *M*=44.6)	HAM-D_{17} ≧8	20 Hz rTMS at left DLPFC	Total 10 daily rTMS sessions Total 10000 pulses	Fast Fourier transform (resting EC: 2-min; EO:2-min; epoch: 300-second)	-Alpha band (8-13 Hz) power changes (post-pre): Responder > Non-responder at prefrontal sites but without site specificity<br>-Negative correlation between alpha power changes and the percent changes in the retardation subscale of HAM-D at F4

Table 3. *(cont.)*

Brain oscillations/ connectivity	Article	Subject and Sample size	Severity of depression	Intervention	Treatment duration	Analytical methods	Results
Alpha oscillations (studies that alpha might change after the treatment)	Yadollahpour et al. (2019)	10 MDD	$HAM\text{-}D_{17}$ as clinical assessment	20 Hz rTMS at left DLPFC	Total 10 daily rTMS sessions	N/A	-FAA (Fp1-Fp2) changes toward right in responders<br>-FAA changes was positive correlated with treatment response
	Olejarczyk et al. (2021)	Group1: 35 MDD (Age: *M*=50.2)<br>Group 2: 77 MDD (Age: *M*=50.0)<br>Group 3: 14 MDD (Age: *M*=55.0)	$HAM\text{-}D_{17} > 18$	1 Hz rTMS at right DLPFC<br>10 Hz rTMS at left DLPFC<br>[e]iTBS at left DLPFC	Group1: One session 120 pulses<br>Group 2: One session 1600 pulses<br>Group 3: One session 600 pulses	Directed Transfer Function (DTF) (resting EC: 10-min; epoch: 30-second)	Fontal alpha oscillations (9-12 Hz) after TMS decreased in group 1 but increase in group 2 but with no impact in group 3

Table 3. *(cont.)*

Brain oscillations/ connectivity	Article	Subject and Sample size	Severity of depression	Intervention	Treatment duration	Analytical methods	Results
Alpha oscillations (studies that alpha might not change after the treatment)	Loo et al. (2001)	18 MDD (Age: *M*=48.0)	HAM-D as clinical assessment	10 Hz rTMS at left DLPFC	Total 10 daily rTMS sessions	N/A (resting: 20-min)	No EEG changes after the treatment
	Spronk et al. (2008)	8 MDD (Age: *M*=42.6)	BDI	10 Hz rTMS at left DLPFC	15-25 sessions	Fast Fourier transform (resting EC:2-min; EO:2-min; epoch: 4-second)	No significant alpha (8-13 Hz) changes have found
	Li et al. (2013)	30 TRD (Age: *M*= 51) 50 healthy controls (Age: *M*= 49, only did MEG resting recording at baseline)	$\text{HAM-D}_{17} \geqq 18$	10 Hz rTMS at left DLPFC	Total 10 daily rTMS sessions Total 16000 pulses	Fast Fourier transform (resting EO: 3-min;	FAA (8-13 Hz) changes cannot distinguish TRD and controls or responders and non-responders

Table 3. *(cont.)*

Brain oscillations/ connectivity	Article	Subject and Sample size	Severity of depression	Intervention	Treatment duration	Analytical methods	Results
Theta oscillations	Heikman et al. (2001)	7 MDD<br>24-46 y/o<br>(Age: *M*=35.1 y/o)	MADRS (*M*=31.8)	[a]ECT	9 times	Fast Fourier transform (resting state: EC: 4-min; EO: 4-min; epoch: 3.4-second)	Increased frontal theta (3-7 Hz) activity after the treatment is related to ECT efficacy
	Valiulis et al. (2012)	45 [b]TRD<br>(Age: *M*=52.2)	$HAM\text{-}D_{17}$, BDI and MADRS as clinical assessment	10 Hz rTMS at left DLPFC (N=23)<br>1 Hz rTMS at right DLPFC (N=22)	Total 10-15 daily rTMS sessions	Fast Fourier transform (resting EC: 10-min; epoch: 2-second	Theta power increased in the central, parietal and occipital regions, as well as across the whole brain after 10-Hz rTMS
	Noda et al. (2013)	25 TRD<br>(Age: *M*=44.6)	$HAM\text{-}D_{17} \geqq 8$	20 Hz rTMS at left DLPFC	Total 10 daily rTMS sessions<br>Total 10000 pulses	Fast Fourier transform (resting EC: 2-min; EO:2-min; epoch: 300-second)	Theta band power significantly increases at seven prefrontal electrode sites after treatment

Table 3. *(cont.)*

Brain oscillations/ connectivity	Article	Subject and Sample size	Severity of depression	Intervention	Treatment duration	Analytical methods	Results
(contradictory results)	Loo et al. (2001)	18 MDD (Age: *M*=48.0)	HAM-D as clinical assessment	10 Hz rTMS at left DLPFC	Total 10 daily rTMS sessions	N/A (resting: 20-min)	No EEG changes after the treatment
	Spronk et al. (2008)	8 MDD (Age: *M*=42.6)	BDI	10 Hz rTMS at left DLPFC	15-25 sessions	Fast Fourier transform (resting state EC:2-min; EO:2-min; epoch: 4-second)	No significant theta changes have found
Theta cordance	Cook et al. (2002)	51 MDD (Age: *M*=41.4 y/o)	$HAM\text{-}D_{17} \geqq 17$	Medication: Fluoxetine; Venlafaxine or placebo	1 week	Fast Fourier transform (resting state: EC; epoch: 20-32 seconds)	Theta cordance decreased after drug treatment in responders
	Bares et al. (2015)	25 MDD (Age: 18-65)	MADRS≧20	1 Hz rTMS at right DLPFC	Total 20 daily rTMS sessions Total 12000 pulses	Fast Fourier transform (resting state EC: 10-min; epoch: 4-second)	Theta cordance (4-8 Hz) decreased after 1 week treatment in responders

Table 3. *(cont.)*

Brain oscillations/ connectivity	Article	Subject and Sample size	Severity of depression	Intervention	Treatment duration	Analytical methods	Results
Theta cordance	Hunter et al. (2018)	20 MDD (Age: M=47.1)	CGI-I and Patient Health Questionnaire (PHQ-9) are as clinical assessments	10 Hz rTMS at left DLPFC	Total 30 daily rTMS sessions Total 90000 pulses	qEEG (resting state EC; epoch:2-second)	Theta cordance (4-8 Hz) in central brain region after 1 week of treatment was positive correlated with the severity of depression at week 6
Fronto-midline theta	Bailey et al. (2018)	50 TRD (Age: M=47.1) 20 controls	HAM-D_{17}, MADRS and BDI-II are as clinical assessments	10 Hz rTMS at left DLPFC	Total 15-40 daily rTMS sessions	Morlet wavelet transform	Responders showed higher working memory related fronto-midline theta power than non-responders at baseline and week 1
	Fitzgerald (2019)	120 MDD	N/A	10 Hz rTMS at left DLPFC	Total 5 daily rTMS sessions	N/A	Responders showed higher working memory related and resting fronto-midline theta power than non-responders at week 1

Table 3. *(cont.)*

Brain oscillations/ connectivity	Article	Subject and Sample size	Severity of depression	Intervention	Treatment duration	Analytical methods	Results
[f]Theta-alpha amplitude modulation	Tsai et al. (2022)	61 TRD (Age: *M*=48.8)	HAM-D_{17} ≧18	10 Hz rTMS (N=20); [b]piTBS (N=19); Sham (N=22) at left DLPFC	Total 10 daily rTMS sessions Total 16000 pulses for rTMS; Total 18000 pulses for piTBS	[c]HHSA (resting state: EC: 5-min; epoch: 10-second)	Theta-alpha amplitude modulation increased after piTBS in responders compared to sham and was positively correlated to improvement of depressive symptoms
Gamma oscillations	Pathak et al. (2016)	5 MDD (Age: N/A)	MADRS	10 Hz rTMS at left DLPFC	Total 20 daily rTMS sessions	Power spectral density (resting state: EC: 6-min/per block; EO:6-min/per block; total 24-min)	Increased in gamma power (30-59 Hz) was positively correlated to improvement of depressive symptoms
	Noda et al. (2017)	31 TRD (Age: *M*=43)	HAM-D_{17} ≧ 10	20 Hz rTMS at left DLPFC	Total 10 daily rTMS sessions Total 10000 pulses	Fast Fourier transform (resting state: EC: 10-min; epoch: 3-min)	Resting gamma power (30-70 Hz) increased at F3 after treatment in all depression. Moreover, the increment was positively correlated with improvement of symptoms.

Table 3. *(cont.)*

Brain oscillations/ connectivity	Article	Subject and Sample size	Severity of depression	Intervention	Treatment duration	Analytical methods	Results
Theta connectivity	Bailey et al. (2019)	42 TRD (Age: *M*=45.9) 21 controls	HAM-D_{17} > 20	10 Hz rTMS at left DLPFC	Total 25-40 daily rTMS sessions	Phase synchronization (weighted phase lagged) (resting state: EC and EO; epoch: 2-second)	Resting theta connectivity (4-8 Hz) widespread increased in responders at week 1
	Bailey et al. (2021)	193 MDD (Age: *M*=43.2)	BDI-II	10 Hz rTMS at left DLPFC or 1 Hz rTMS at right DLPFC	Mean 20.9 sessions	Phase synchronization (weighted phase lagged) (resting state: EC and EO: total 2-min; epoch: 2-second)	The results could not replicate the study in Bailey et al. (2019)
Beta connectivity	Kito et al. (2017)	14 TRD (Age: *M*=43.2)	HAM-D_{17} > 18	10 Hz rTMS at left DLPFC	Total 20 daily rTMS sessions	Lagged nonlinear connectivity analysis in sLORETA (resting state: EC: 5-min; epoch: 5-second)	Increased beta connectivity (19-24.5 Hz) between left DLPFC and limbic region after the treatment in all participants

Table 3. *(cont.)*

Brain oscillations/ connectivity	Article	Subject and Sample size	Severity of depression	Intervention	Treatment duration	Analytical methods	Results
Gamma connectivity	Pathak et al. (2016)	5 MDD (Age: N/A)	MADRS	10 Hz rTMS at left DLPFC	Total 20 daily rTMS sessions	Coherence	Responders showed the reduction of gamma connectivity (30-59 Hz) between left DLPFC and sgACC
	Bailey et al. (2018)	50 TRD (Age: *M*=47.1) 20 controls	MADRS	10 Hz rTMS at left DLPFC	Total 15-40 daily rTMS sessions	Phase synchronization (weighted phase lagged)	Responders showed enhanced fronto-parietal gamma connectivity (30-45 Hz) at week1 during resting state as well as working memory task
Theta-gamma coupling	Noda et al. (2017)	31 TRD (Age: *M*=43)	$HAM\text{-}D_{17} \geqq 10$	20 Hz rTMS at left DLPFC	Total 10 daily rTMS sessions Total 10000 pulses	PAC	Resting theta-gamma coupling increased at C3 and T3 after treatment in all depression

Note

The order of the articles in this table was summarized by the following rules: First is the study result which showed a similar pattern. Second is the state, e.g., resting state. Third is the year of the publications.

[a] Electroconvulsive therapy (ECT)

[b] Prolonged intermittent theta burst stimulation (piTBS), which is three times of pulses of the standard parameters (600 pulses)

[c] Holo-Hilbert Spectral Analysis (HHSA)
